# Basic psychological needs, academic self-efficacy, self-leadership, career adaptability, and life-satisfaction: Data-set from Turkish university students

**DOI:** 10.1016/j.dib.2022.107834

**Published:** 2022-01-15

**Authors:** Fatma Uslu Gülşen, Ezgi Ekin Şahin

**Affiliations:** Mersin University, Faculty of Education, Mersin, Turkey; Anadolu University Counseling Center, Eskişehir, Turkey

**Keywords:** Career adaptability, College experience, Emotional experience, Educational psychology, Turkey

## Abstract

This data article provides data set from variables related to career adaptability, academic self-efficacy, self-leadership, basic psychological needs, and life satisfaction among college students in Turkey. The data were collected with an online survey from May to July 2021, from a sample of 404 college students of several universities in Turkey. Among the participants, there were 272 (67.3%) females and 132 (32.7%) males, with an average age of 22.10 (SD=3.13). Among the participants, 86 (21.3%) were freshmen, 49 (12.1%) were sophomores, 149 (36.9%) were juniors, and 120 (29.7%) were seniors from different faculties. They completed General Need Satisfaction Scale, Academic Self-Efficacy Scale, The Abbreviated Self-Leadership Questionnaire, Career Adapt-Abilities Scale–Short Form, and Satisfaction with Life Scale. The data was used for quantitative analysis through structural equation modeling to test the hypothetical model with latent variables. The researchers may be able to use the data to explore the relationships between career adaptability, basic psychological needs, academic self-efficacy, self-leadership, and life satisfaction of college students.

## Specifications Table

**Table:** 

Subject	Higher Education
Specific subject area	Education Psychology, Education Management,
Type of data	Table Figure Data Excel
How the data were acquired	Data was collected online survey by way of Google Forms (a web-based survey platform). Informed consent was obtained. The questionnaires are provided as in the repository. All valid samples were processed with SPSS 26.0 and LISREL 8.7.software to conduct Cronbach's Alpha, SEM analysis.
Data format	Raw Analyzed
Description of data collection	Data collection started on May 2021 and was closed on July 2021. All the participants were informed of the study purpose and provided consent to participate. The survey link was shared several lecturers working at different states of Turkey and they shared the link of survey with their students. Participants were also encouraged to share the link to reach more college students through the snowball sampling method.
Data source location	• Mersin, Eskişehir, Çankırı, İstanbul, Adana, Antalya • Turkey
Data accessibility	Repository name: Mendeley Data Data identification number: 10.17632/b5yc9sw7y9.5 Direct URL to data: https://data.mendeley.com/datasets/b5yc9sw7y9/5

## Value of the Data

• The data set provides information about the mediating role of self-leadership in the relationships between basic psychological needs and career adaptability and academic self-efficacy and career adaptability among college students.
• The data set provide researchers a better understanding between self-leadership, basic psychological needs, academic self-efficacy, career adaptability, and life satisfaction of college students.
• The data can be used by the researchers who want to conduct further cross-cultural studies on the career adaptability of college students.
• The Structural Equation Model (SEM) and other analyses can be applied by this data.
• Analyses derived from the data could assist universities in preparing career counseling services to create career awareness of college students.
• Exploring career adaptation perception of college students may enable an evaluation of the higher education system's facilities such as student social support and career counselling services.

## 1 Data Description

The data set presents results from an online survey including a demographic form and five questionnaires (i) General Need Satisfaction Scale-GNSS [1], (ii) Academic Self-Efficacy Scale- ASES [2], (iii) The Abbreviated Self-Leadership Questionnaire-TASLQ [3], (iv) Career Adapt-Abilities Scale–Short Form-CASSF [4], and (v) Satisfaction with Life Scale-SWLS [5]. Demographic information of the population includes questions about age, gender, grade level, university type (public, private) and majors of the population. The demographic profile of participants in frequencies and proportions was presented in Table 1. GNSS measures fulfillment of basic psychological needs in three domains as autonomy, competence, relatedness with 21 items. ASES evaluates the self-efficacy level of college students in the academic domain with one dimension and seven items. TASLQ includes three factors as behavior awareness and volition, task motivation, constructive cognition in order to measure the general self-leadership construct with nine items. CASSF measures career adaptation of undergraduate students with four factors named concern, control, curiosity, and confidence with 12 items. SWLS is unidimensional with five items. The data was obtained the Turkish forms of all the scales. The questionnaires are provided as in the repository. The raw data set is presented at https://data.mendeley.com/datasets/b5yc9sw7y9/5

**Table 1:** Demographic profile of participants (*N* = 404).

Demographic		Frequency	Proportion (%)
Gender	Female	272	67,30
	Male	132	32,70
Age	18	15	3,71
	19	26	6,44
	20	47	11,63
	21	88	21,78
	22	102	25,25
	23	80	19,80
	24	18	4,46
	25	8	1,98
	>25	20	4,95
Grade level	First-year	86	21,3
	Second-year	49	12,1
	Third-year	149	36,9
	Fourth-year	120	29,7
University Type	State	34	70,83
	Private	14	29,17
Students numbers according to university type	State	377	93,3
	Private	27	6,7
Majors	Education	177	43,8
	Literature	121	30
	Engineering	39	9,7
	Economics, Business and Management	17	4,2
	Architecture	10	2,5
	Other majors	40	9,90

The descriptive statistics (means and standard deviations) of all variables were displayed in Table 2 and correlations among variables were presented in Table 3.

**Table 2:** Descriptive results of participants’ responses.

					Mean			
Variables	Items	Range	Min	Max	Statistics	Std. Error	Std. Deviation	Variance
Academic Self-efficacy	ACSE1	3	1	4	3,15	,032	,643	,413
	ACSE2	3	1	4	3,29	,031	,630	,397
	ACSE3	3	1	4	3,24	,032	,639	,409
	ACSE4	3	1	4	2,78	,038	,762	,581
	ACSE5	3	1	4	2,30	,041	,832	,692
	ACSE6	3	1	4	2,75	,038	,768	,590
	ACSE7	3	1	4	2,81	,045	,901	,811
Basic Psychological Needs	BPN1	6	1	7	5,15	,080	1,605	2,577
	BPN2	6	1	7	5,19	,064	1,285	1,650
	BPN3	6	1	7	3,89	,088	1,760	3,099
	BPN4	6	1	7	3,60	,089	1,780	3,168
	BPN5	6	1	7	5,64	,054	1,081	1,169
	BPN6	5	2	7	5,91	,052	1,038	1,077
	BPN7	6	1	7	4,11	,095	1,910	3,648
	BPN8	6	1	7	5,32	,074	1,495	2,235
	BPN9	6	1	7	5,42	,066	1,324	1,752
	BPN10	6	1	7	4,47	,089	1,785	3,188
	BPN11	6	1	7	3,52	,080	1,599	2,558
	BPN12	6	1	7	5,39	,061	1,226	1,504
	BPN13	6	1	7	5,51	,066	1,321	1,744
	BPN14	6	1	7	5,36	,064	1,296	1,680
	BPN15	6	1	7	3,55	,087	1,751	3,067
	BPN16	6	1	7	3,77	,099	1,988	3,953
	BPN17	6	1	7	5,18	,074	1,494	2,231
	BPN18	6	1	7	5,15	,085	1,704	2,904
	BPN19	6	1	7	4,65	,090	1,808	3,270
	BPN20	6	1	7	4,56	,088	1,774	3,145
	BPN21	6	1	7	5,62	,053	1,072	1,150
Self-leadership	SELFLD1	4	1	5	3,93	,039	,781	,611
	SELFLD2	4	1	5	4,07	,043	,860	,740
	SELFLD3	4	1	5	3,84	,057	1,148	1,318
	SELFLD4	4	1	5	4,11	,038	,761	,579
	SELFLD5	4	1	5	4,12	,039	,784	,614
	SELFLD6	4	1	5	4,06	,037	,749	,562
	SELFLD7	4	1	5	4,07	,038	,757	,573
	SELFLD8	4	1	5	3,85	,053	1,057	1,118
	SELFLD9	4	1	5	4,07	,036	,722	,521
Career Adaptability	CADAPT1	4	1	5	3,91	,049	,994	,988
	CADAPT2	4	1	5	3,71	,048	,960	,922
	CADAPT3	4	1	5	3,92	,047	,948	,899
	CADAPT4	4	1	5	4,09	,045	,908	,824
	CADAPT5	4	1	5	4,40	,038	,757	,573
	CADAPT6	4	1	5	3,96	,050	,997	,994
	CADAPT7	4	1	5	3,93	,047	,948	,898
	CADAPT8	4	1	5	4,24	,040	,798	,637
	CADAPT9	4	1	5	4,03	,044	,875	,766
	CADAPT10	4	1	5	4,35	,037	,748	,560
	CADAPT11	4	1	5	4,00	,043	,869	,754
	CADAPT12	4	1	5	4,02	,044	,880	,774
Life-satisfaction	LIFESAT1	4	1	5	2,97	,053	1,067	1,138
	LIFESAT2	4	1	5	2,74	,054	1,082	1,172
	LIFESAT3	4	1	5	3,15	,057	1,149	1,321
	LIFESAT4	4	1	5	2,99	,053	1,072	1,149
	LIFESAT5	4	1	5	2,40	,062	1,249	1,561

**Table 3:** Correlations among the variables

Variables	1.	2.	3.	4.	5.
1. Academic self-efficacy	-				
2. Basic psychological needs	.35	-			
3. Self-leadership	.40	.43	-		
4. Career adaptability	.51	.53	.53	-	
5. Life-satisfaction	.22	.44	.25	.33	-

*Note*. All the correlations are significant at .01 level.

## 2 Experimental Design, Materials and Methods

The data were collected with an online survey from May to July 2021, from a sample of 404 college students of several universities in Turkey. The online survey distributed lecturers working several universities in Turkey and asked to share the survey link with their students via WhatsApp groups. It was also asked to these participants to share the URL with their friends from different universities in Turkey. The survey was closed in the last week of July when no responses were collected for one week. Informed consent which include statements about the use of the information for academic purposes, and participation was voluntary was obtained from all participants. The anonymity of participants was ensured in the survey which did not included any personal information. SEM with latent variables was used to test the hypothetical model, LISREL 8.7 was used to conduct the analysis. T values were used to evaluate the path coefficients in the model. The final model was determined using the Chi-square difference test (x^2^). For the mediation test, a nested model method was also applied.

## Ethics Statement

The ethics committee of Anadolu University approved the surveys before implementation (Protocol Number 81783), respondents provide consent to use the information for academic purposes, and participation was totally voluntary.

## CRediT Author Statement

**FUG:** Conceptualization, Investigation, Writing – Reviewing and Editing; **EEŞ:** Conceptualization, Data Curation, Methodology, Software, Visualization, Formal Analysis.

## Declaration of Competing Interest

The authors declare that they have no known competing financial interests or personal relationships that could have appeared to influence the work reported in this paper.
